# Photoswitching-Free FRAP Analysis with a Genetically Encoded Fluorescent Tag

**DOI:** 10.1371/journal.pone.0107730

**Published:** 2014-09-18

**Authors:** Tatsuya Morisaki, James G. McNally

**Affiliations:** Fluorescence Imaging Group, National Cancer Institute, National Institutes of Health, Bethesda, Maryland, United States of America; University of Michigan, United States of America

## Abstract

Fluorescence recovery after photobleaching (FRAP) is a widely used imaging technique for measuring protein dynamics in live cells that has provided many important biological insights. Although FRAP presumes that the conversion of a fluorophore from a bright to a dark state is irreversible, GFP as well as other genetically encoded fluorescent proteins now in common use can also exhibit a reversible conversion known as photoswitching. Various studies have shown how photoswitching can cause at least four different artifacts in FRAP, leading to false conclusions about various biological phenomena, including the erroneous identification of anomalous diffusion or the overestimation of the freely diffusible fraction of a cellular protein. Unfortunately, identifying and then correcting these artifacts is difficult. Here we report a new characteristic of an organic fluorophore tetramethylrhodamine bound to the HaloTag protein (TMR-HaloTag), which like GFP can be genetically encoded, but which directly and simply overcomes the artifacts caused by photoswitching in FRAP. We show that TMR exhibits virtually no photoswitching in live cells under typical imaging conditions for FRAP. We also demonstrate that TMR eliminates all of the four reported photoswitching artifacts in FRAP. Finally, we apply this photoswitching-free FRAP with TMR to show that the chromatin decondensation following UV irradiation does not involve loss of nucleosomes from the damaged DNA. In sum, we demonstrate that the TMR Halo label provides a genetically encoded fluorescent tag very well suited for accurate FRAP experiments.

## Introduction

Fluorescence recovery after photobleaching (FRAP) is a technique widely used to analyze protein dynamics in live cells [1], [2]. In FRAP, a sub-region of a live cell expressing a fluorescently labeled protein of interest is subjected to a brief, high intensity light pulse designed to induce fluorophores into a permanent dark state. Fluorescence in this photobleached zone recovers as time passes due to the inward migration of surrounding fluorescently labeled proteins. By plotting this recovery in fluorescence intensity within the photobleached zone as a function of time, FRAP recovery curves can be generated. Steeper FRAP recovery curves indicate higher mobility of the protein under study. These protein dynamics can then be analyzed qualitatively by comparing differences in FRAP recovery curves, for example before and after a certain stimulus or between a wild-type and a mutant. Protein dynamics can also be analyzed quantitatively by fitting the FRAP recovery curves with mathematical models. Such quantitative analysis yields various parameters about protein dynamics, including diffusion constants, on and off rates of binding and the fraction of bound proteins [3]. Since FRAP is easy to perform and the resultant data are intuitive, it has been widely used to investigate the dynamics of proteins inside live cells, and has provided many important biological insights [4]–[6].

However, it is now known that one assumption commonly made in FRAP experiments is not always valid, and as a result severe artifacts can arise [7]–[10]. FRAP presumes that only one pathway exists for fluorophore conversion, namely illumination causes bright fluorophores to enter into a permanent dark state and become “bleached” [11]. However, in many cases, illumination can also cause fluorophores to enter into a transient dark state that can then revert back to the bright state [7]–[10], [12]–[15]. This process of switching between a bright and a transient dark state is known as “photoswitching”. Photoswitching can introduce a number of severe artifacts into FRAP experiments. Hence ignoring the photoswitching pathway of a fluorophore in a FRAP experiment can lead to erroneous conclusions about various biological phenomena [7]–[10].

Photoswitching artifacts arise in FRAP because FRAP involves time-lapse imaging both before and after the photobleach, and time-lapse imaging is very sensitive to photoswitching [7]. Sinnecker et al. [7] have shown that fluorophores exhibit a bi-phasic decay which reflects the two different dark states available during time-lapse. The faster phase of the biphasic decay reflects rapid entry of fluorophores into the transient dark state. As the transient dark state becomes populated, fluorophore reversion to the bright state increases, eventually establishing an equilibrium in photoswitching. After this photoswitching equilibrium is reached, then the second slower phase of the biphasic decay dominates reflecting irreversible loss of fluorophores to the permanent dark state (known as observational photobleaching). This photoswitching equilibrium can be perturbed during time-lapse imaging by alterations in either the excitation intensity or temporal sampling rate, both of which typically happen in FRAP experiments. It is now recognized that this photoswitching behavior can disrupt FRAP experiments in at least four different ways [7]–[10].

First, photoswitching can give rise to a large artifactual fast component in a FRAP [7], [9]. Mueller et al. found that the true fast component of a GFP-tagged histone H2B could be overestimated by as much as 60 fold. This artifact arises because the high intensity illumination of the intentional photobleach disturbs the photoswitching equilibrium by driving a much larger fraction of fluorophores into the transient dark state than had been achieved during pre-bleach time-lapse imaging. Upon return to post-bleach time-lapse imaging, the now overpopulated dark state generates a rapid reversion of many fluorophores to the bright state, producing an apparent fluorescent recovery that mimics high mobility. This artifact is especially pronounced for proteins with slow FRAP recoveries because many molecules of these proteins remain in the photobleached region long enough for the photoswitching reversion to occur, whereas for proteins with fast recoveries a larger fraction of molecules can diffuse out of the photobleached region before the reversion occurs.

Second, photoswitching can give rise to artifactually faster FRAP curves throughout the entire recovery [10]. Daddysman and Fecko showed that the intentional photobleach can cause the photobleached region to reach the slower phase of the biphasic decay sooner than surrounding regions of the same or other cells, yet those other regions are typically used to correct the FRAP recovery for observational photobleaching in the photobleached region. This can lead to overcorrection of the FRAP curve if these other regions are still in the fast phase of the biphasic decay while the photobleached region has reached the slow phase of the biphasic decay.

Third, both Sinnecker and Bancaud have noted that photoswitching can cause a discontinuous jump in the FRAP recovery curve if the temporal sampling rate is changed during the recovery [7], [8]. This arises because the altered temporal sampling disturbs the photoswitching equilibrium. For example, a reduction in the sampling rate will drive fewer molecules into the transient dark state and so the reversion to the bright state is temporarily too large producing an upward jump in the FRAP curve.

Fourth, Mueller et al. observed that photoswitching can cause the first image after the photobleach to be dimmer than it should be whenever the microscope software fails to maintain an equal spacing of images at the time of the photobleach (a common feature of many commercial instruments) [9]. They found that this did not significantly change the shape of the FRAP recovery curve, but it did radically alter the measured profile of the photobleach. Therefore a correction procedure is necessary to perform a quantitative fit to the FRAP curve because fits rely on accurate determination of the photobleach profile [16].

Thus photoswitching can confound FRAP analysis in multiple ways. These can have serious biological consequences such as the erroneous identification of anomalous diffusion [10] or the overestimation of the fast component of a protein [7], [9]. In an attempt to undo these effects of photoswitching on FRAP, two different groups have proposed mathematical correction procedures [9], [10]. However, the simplest approach to deal with photoswitching would be to use a fluorophore that minimizes the effect. Unfortunately, GFP as well as other fluorescent proteins now in common use can exhibit considerable photoswitching [7], [9].

In this study, we found that an organic fluorophore tetramethylrhodamine (TMR) can overcome the problems caused by photoswitching. First we showed that TMR exhibits virtually no photoswitching behavior in live cells under typical imaging conditions for FRAP experiments. We then showed that FRAP experiments performed with TMR exhibit none of the four photoswitching artifacts previously reported in FRAP.

Finally we applied this photoswitching-free FRAP procedure to test whether UV-induced DNA damage causes release of nucleosomes from the damaged DNA. The nucleosome, which consists of eight core histone proteins wrapped with DNA, is the basic structural unit of the eukaryotic genome. As such, nucleosomes play a key role in the regulation of many nuclear processes, including gene transcription and DNA replication. Nucleosomes regulate these processes by altering the accessibility of proteins to specific regions of the DNA.

For example, it is known that DNA damage causes chromatin decondensation which facilitates DNA repair by increasing access of various factors to the damaged DNA [17]–[19]. This increased access could occur by at least two different processes: wholesale loss of nucleosomes from the DNA or more rapid exchange of individual histone proteins with the nucleosome. Increased exchange of histone proteins has indeed been detected in FRAP experiments [19], but determining whether nucleosome loss also occurs has been problematic. Nucleosome loss would be detected in a FRAP experiment as a larger free fraction of histone proteins. However this has been very difficult to measure because the free fraction of histones within cells is very small and so is easily obscured by the photoswitching fraction of a FRAP recovery curve. To detect a change in the free fraction of a histone protein, we performed FRAP of H2B-Halo-TMR within cells before and after UV irradiation. We found no significant difference in the free fraction suggesting that chromatin decondensation after DNA damage does not involve loss of nucleosomes from the damaged DNA.

## Results

Sinnecker et al. [7] have suggested that photoswitching of CFP and YFP can be identified and quantified by the measurement of fluorescent intensity during time-lapse imaging. They argued that when photoswitching occurs, the intensity time course should exhibit a biphasic decay, namely a rapid decay followed by a slower decay.

We extended these observations to GFP by imaging live cells expressing a GFP-tagged histone H2B (H2B-GFP). We performed all of our experiments in vivo since the photoswitching characteristics of fluorophores are very sensitive to the environment (such as pH). By working with live cells, we could characterize photoswitching under the same conditions used in a FRAP experiment. By working with H2B, we could readily distinguish photoswitching behavior from fluorophore redistributions, since the photoswitching behavior is on a time scale of seconds while the H2B FRAP recovery is on a time scale of hours with at most a 1% freely diffusible fraction [9], [20].

**Figure 1:**
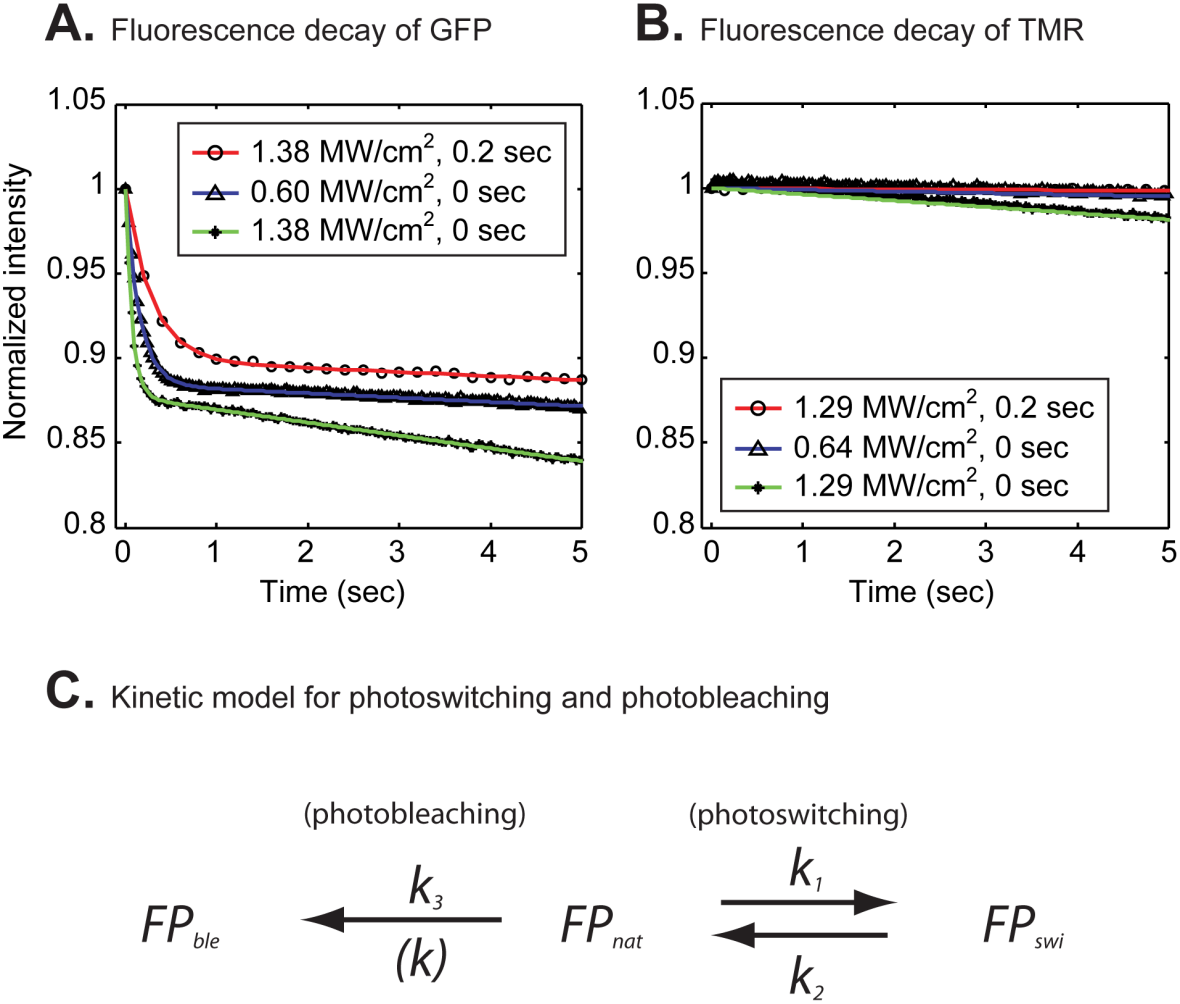
Fluorescent decay curves of H2B-GFP (A) and H2B-Halo-TMR (B) during time lapse imaging of entire nuclei in live cells. The GFP tag exhibited a bi-phasic decay with three different combinations of the laser power and delay time between images (A). Colored curves indicate fits to the data with a bi-exponential decay model. According to the model (C), fluorescent molecules 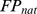 can convert at a rate 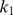 into a photoswitched dark state 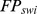 and then revert to the fluorescent state at a rate 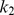. Fluorescent molecules can also bleach irreversibly to a dark state  at a rate 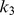. In contrast to the GFP tag, the TMR-Halo tag exhibited a monophasic decay that was well fit by a single exponential (colored curves) (B). Fitting parameters are shown in Table 1.

To measure the decay kinetics of GFP during time lapse, we used laser intensities suitable for the recovery phase of a FRAP and imaged the entire cell nucleus at each time point. We collected time-lapse images varying both laser power and frame rates. The resultant fluorescent decay curves were biphasic (Fig. 1A) and poorly fit by a single exponential, but well fit by the double exponential model for photoswitching proposed by Sinnecker et al. [7] (Fig. S1A and Table S1A in Supporting Materials S1). The model is based on the interconversion scheme shown in Fig. 1C, where 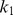 and 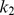 are the forward and backward rate constants that define conversion and reversion respectively from a fluorescent dark state, and 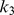 is the forward rate constant that defines conversion into an irreversibly photobleached state (See Supporting Materials S1 Sect. 2 for details). Consistent with Sinnecker et al. [7], we also found that these rate constants increased with increasing laser power and decreased with slower temporal sampling rates (Table 1).

**Table 1:** Photoconversion rates of GFP obtained from fits to fluorescent decay curves (estimates±95% confidence intervals).

Laser power (MW/cm^2^)	Delay (sec)	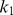 (sec^−1^)	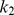 (sec^−1^)	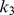 (sec^−1^)
1.38	0	1.7±0.029	12±0.22	0.010±0.00012
0.6	0	0.75±0.010	5.7±0.081	0.0033±0.00013
1.38	0.2	0.36±0.011	3.2±0.12	0.0030±0.00040

The double exponential model for photoswitching was necessary to explain the GFP fluorescent decay. Photoconversion rates increased with increasing laser power and decreased with slower temporal sampling rates as previously described [7].

To evaluate whether TMR exhibited similar behavior we performed comparable time-lapse measurements on H2B labeled with TMR. This was done by fusing H2B with the genetically encoded HaloTag which can be covalently labeled by the TMR-HaloTag ligand (see Supporting Materials S1 Sect. 3 for structural formula) [21]. For a fair comparison with H2B-GFP, we first determined what laser powers were required to produce comparable intensities from either GFP or TMR (see Methods and Supporting Materials S1 Sect. 4), and then used the equivalent laser intensity to perform the time-lapse imaging of H2B-Halo-TMR. Second, we performed time-lapse imaging of TMR for a long enough time period such that the TMR fluorescent decay was comparable to GFP. This required much longer imaging of TMR compared to GFP. Even under these conditions, in contrast to H2B-GFP, the H2B-Halo-TMR fluorescence decay appeared almost linear. However, by applying the Akaike information criterion [22] (see Supporting Materials S1 Sect. 1 for details), we found that the TMR time lapse decay was best described by a single exponential, rather than either a line or a double exponential (Fig. 1B, Table 2, Fig. S1B and Table S1B in Supporting Materials S1). These observations indicate that TMR behaves quite differently from GFP, and in particular suggest that TMR may have little or no photoswitching behavior under typical FRAP imaging conditions. Thus we decided to directly test whether the various photoswitching artifacts reported in FRAP experiments could be reduced or even eliminated by the TMR-HaloTag, thereby obviating the need for complex mathematical correction procedures.

**Table 2:** Photobleaching rates of TMR obtained from fits to fluorescent decay curves (estimates±95% confidence intervals).

Laser power(MW/cm^2^)	Delay (sec)	*k* (sec^−1^)
1.29	0	0.0079±0.000018
0.64	0	0.0020±0.0000014
1.29	0.2	0.0010±0.0000020

Unlike GFP, the single exponential model for simple photobleaching was sufficient to explain the TMR fluorescent decay.

**Figure 2:**
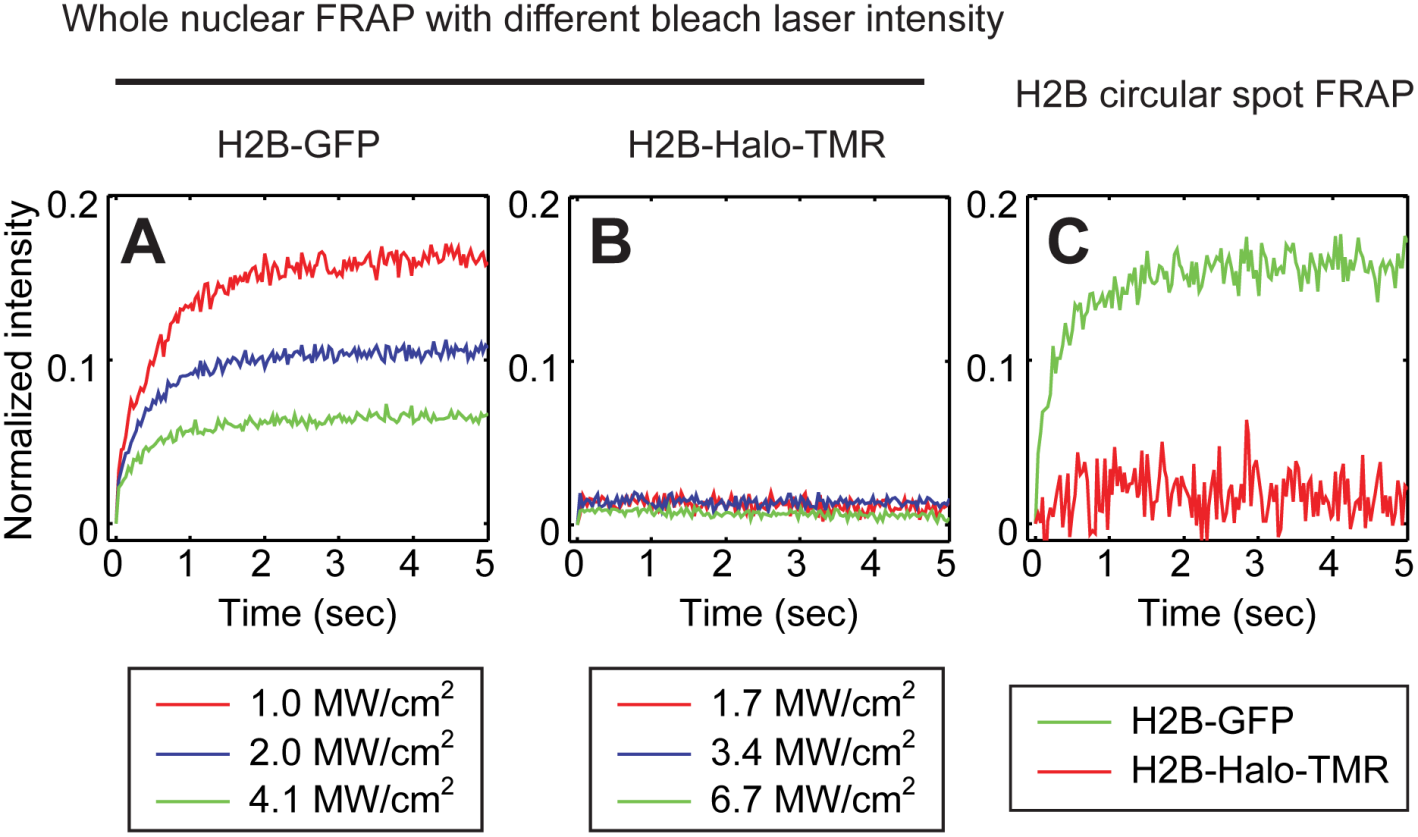
Artifacts in FRAP arising from the strength of the intentional photobleach. The entire nucleus containing either H2B-GFP or H2B-Halo-TMR was bleached to eliminate any conventional fluorescence recovery by influx of unbleached fluorescence, and then whole nuclear intensity was measured. The data were normalized with the pre-bleach intensity set to 1 and the post bleach intensity set to 0. Artifactual recoveries were observed with the GFP tag (A) but not the TMR-Halo tag (B). Consistent with previous studies [9], the relative size of the recovery with the GFP-tag increased with decreasing strength of the photobleach (colored curves in A). No such dependence was seen with the TMR-Halo tag (colored curves in B). Comparable effects were seen when performing a conventional spot FRAP (0.5 µm radius) with H2B exhibiting a fast component of ∼15% with the GFP-tag and ∼1% with the TMR-Halo tag (C). The TMR-HaloTag fast component of 1% for H2B is consistent with a previous study which predicted a 1% fast component after mathematical correction for photoswitching in FRAP of H2B-GFP [9]. Note that the fast component was estimated by finding the point where the change in slope is minimized in the measured curve.

The first, and the most obvious artifact arising from photoswitching in FRAP, is the appearance of a large artifactual fast component introduced by the high intensity illumination during the intentional photobleach [7], [9]. We confirmed that such a fraction could arise with GFP by performing whole nuclear bleaches of live cells expressing H2B-GFP. A whole nuclear bleach eliminates the possibility of any influx of unbleached H2B-GFP since H2B fluorescence is restricted to the nucleus. Therefore, ideally we should not observe any recovery. Nevertheless, we found that whole nuclear bleaches of H2B-GFP gave rise to fluorescent recoveries (6.5–16%) that increased as the laser power used for the whole nuclear photobleach was reduced (Fig. 2A). This is consistent with previous studies and confirms the possibility of significant photoswitching artifacts for GFP FRAPs [9]. To assess if similar artifacts would arise with the TMR-HaloTag we performed whole nuclear bleaches on H2B-Halo-TMR using a laser power that yielded the equivalent bleach depth as the GFP whole nuclear photobleach (Fig. S5 in Supporting Materials S1). In contrast to H2B-GFP, we found virtually no fluorescent recovery when whole nuclear bleaches were performed on H2B-Halo-TMR regardless of the laser power (Fig. 2B, see also Movie S1 for a representative movie of the whole nuclear photobleach). This suggests that the intentional photobleach does not introduce a photoswitchable fraction in TMR-HaloTag.

As a more direct test, we also performed conventional FRAP experiments of H2B in live cells with a bleach spot of 1.5 µm in diameter in a nucleus of ∼15 µm diameter. Under the photobleach conditions used, H2B-GFP yielded an apparent fast component of 15.8±2.4% followed by a much slower recovery rate that reflects the bound fraction of H2B and its exchange rate with chromatin (Fig. 2C). In contrast, H2B-Halo-TMR yielded a fast component of 1.6±1.2%, which is equivalent to the previously measured fast component of H2B-GFP after mathematical correction for photoswitching [9] (Fig. 2C, see also Movie S2 for a representative FRAP movie). This difference in the fast component was the key difference between the GFP and TMR curves as measurement of the FRAP recoveries over a longer time period showed that the recovery rates over this time scale were similar (Fig. S6 in Supporting Materials S1). We conclude that the TMR-HaloTag label virtually eliminates the artifactual fast component that can arise due to the intentional photobleach of a photoswitchable fluorophore such as GFP.

**Figure 3:**
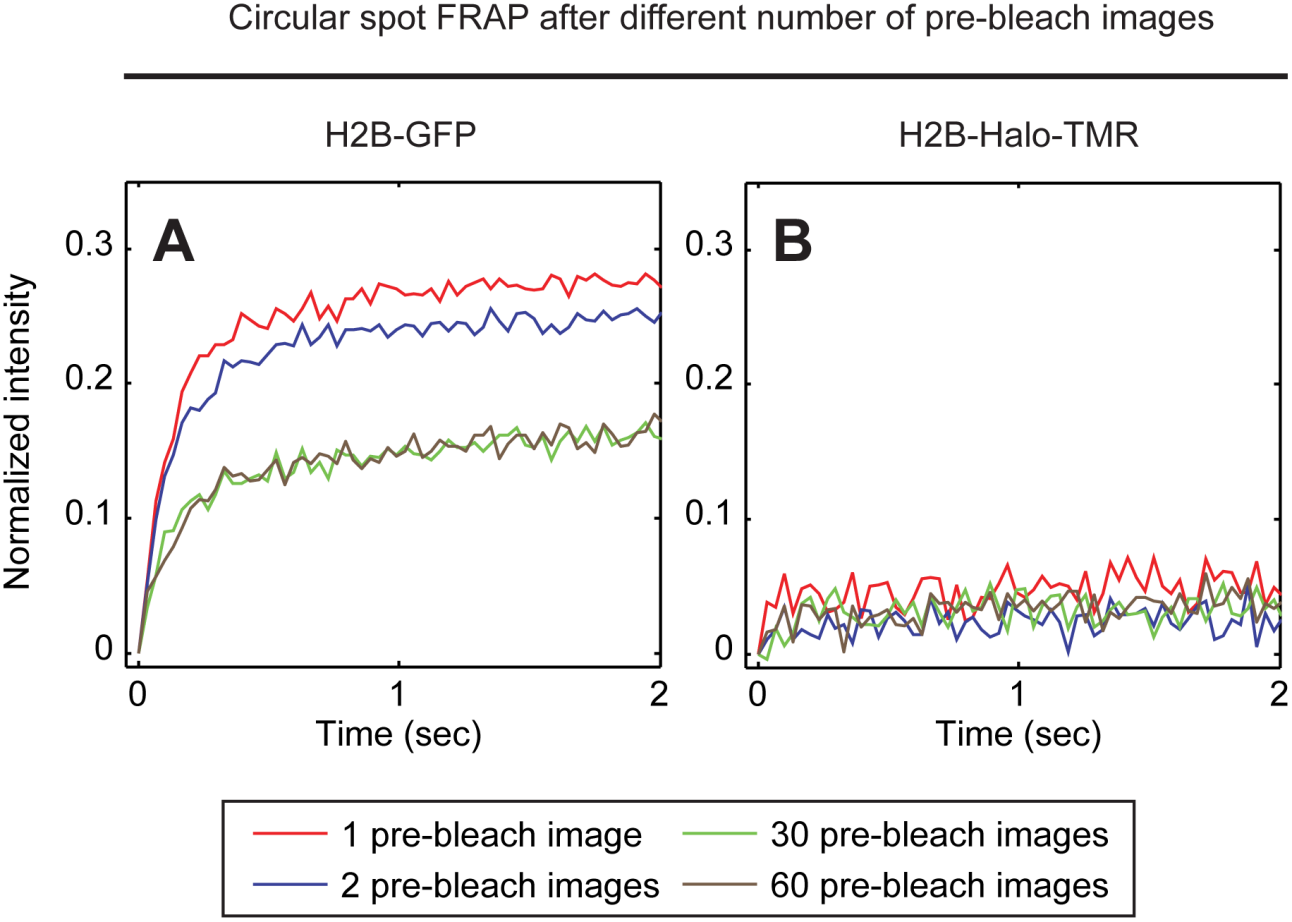
Artifacts in FRAP arising from the timing of the intentional photobleach. Spot FRAPs of H2B tagged with either GFP or TMR-Halo were performed varying only the number of pre-bleach images collected (1, 2, 30, 60). The intensity of the photobleach was kept constant at the level used in Fig. 2C. This yielded FRAP curves that were equivalent to those in Fig. 2C, as long as the number of pre-bleach images was 30 or 60 (60 were used in Fig. 2C). However, when only one or two pre-bleach images were acquired the size of the artifactual fast component increased (A). In contrast, with the TMR-HaloTag no dependence on the timing of the intentional photobleach was seen, with a fast component size of ∼1% in all cases (B).

The second type of photoswitching artifact was reported by Daddysman and Fecko [10]. They found that the correction procedure for observational photobleaching could lead to overcorrected FRAP curves. We confirmed this artifact by performing FRAP of H2B-GFP with different numbers of prebleach images (namely either 1, 2, 30 or 60 pre-bleach images). We then corrected for observational photobleaching by using a control curve measured from a time-lapse imaging sequence of the same cell performed before the photobleach. Following this correction, we found that the FRAP curves obtained after 60 or 30 pre-bleach images were identical, whereas the curves obtained after 2 or 1 pre-bleach images were progressively faster (Fig. 3A). This shows that performing the photobleach at progressively earlier phases of the photoswitching equilibrium process leads to progressive overcorrection of the FRAP recovery when GFP is used. We then repeated these experiments using the H2B-Halo-TMR. Here we found no differences among the four FRAP curves after correction for observational photobleaching regardless of the number of pre-bleach images acquired (Fig. 3B). These results demonstrate that the TMR-HaloTag label is not sensitive to the timing of the intentional photobleach and the resultant artifacts introduced by the correction for observational photobleaching.

The third photoswitching artifact in FRAP was reported by both Sinnecker et al. and Bancaud et al. [7], [8]. They found that a change in temporal sampling rate anywhere during the recovery phase of a FRAP can introduce a “blip” in the recovery curve caused by the shift of the photoswitching equilibrium. This is unfortunate because it is often desirable to change sampling rates since FRAP curves typically exhibit a fast recovery phase where higher temporal sampling is justified followed by a slow recovery phase where lower temporal sampling should suffice and also protect the sample against observational photobleaching. However, with most fluorophores this must be avoided due to the photoswitching artifact.

**Figure 4:**
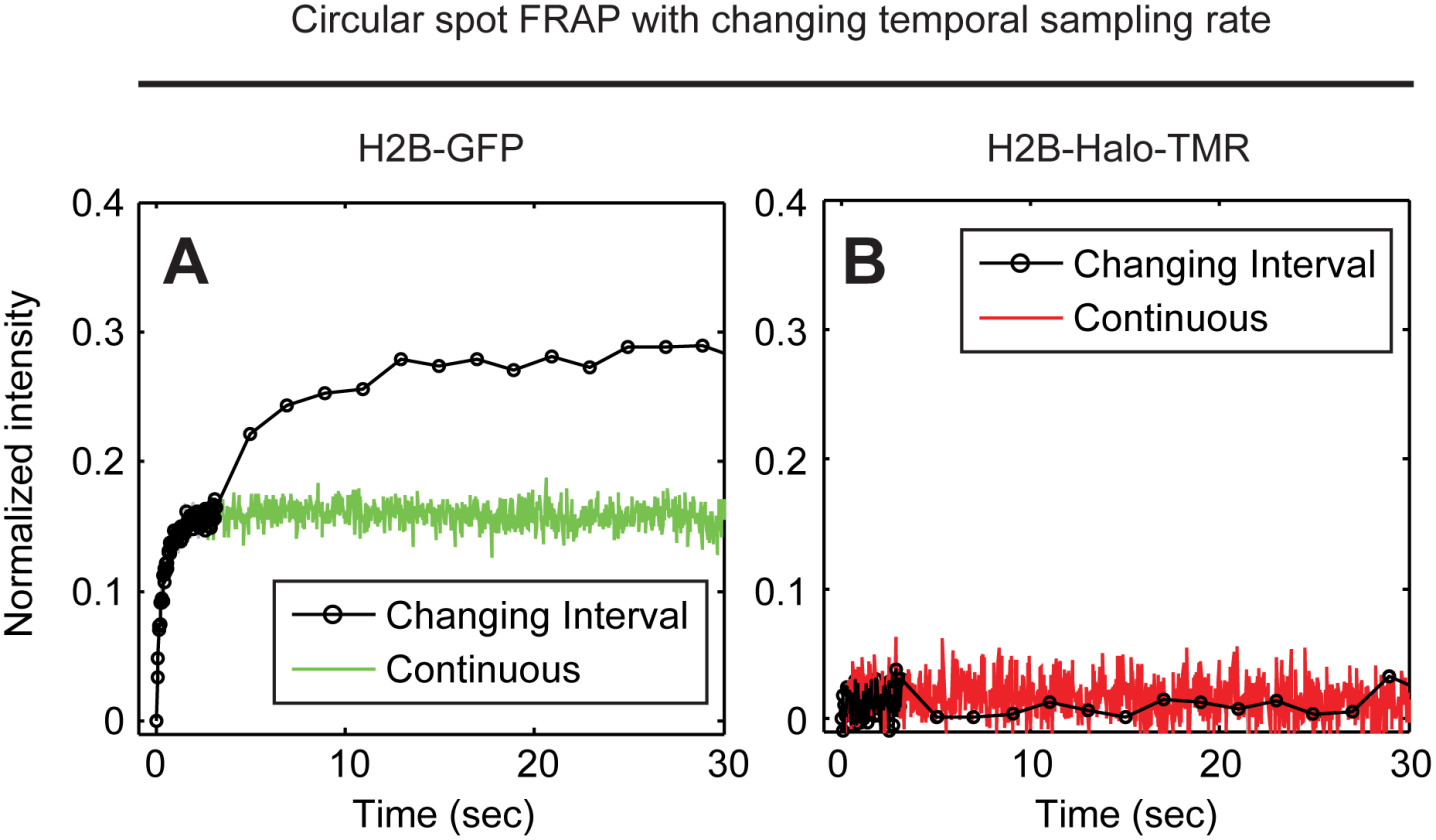
Artifacts in FRAP arising from alteration in the temporal sampling rate during the recovery phase. H2B spot FRAP was performed as in Fig. 2C but now the temporal sampling rate was changed such that data after the photobleach were collected with no delay between images for the first 3 s after the photobleach and then with an interval of 2 s between images at the 3 s time point after the photobleach. With a constant sampling rate, the artifactual fast fraction of ∼15% for the GFP tag is equivalent to that in Fig. 2C (green curve, A), whereas with a change in temporal sampling rate the FRAP curve jumps upward leading to an even larger artifactual fast fraction (black curve, A). In contrast, the TMR-HaloTag shows no such effect with the red and black curves overlapping (B).

We confirmed the presence of this artifact by performing FRAPs of H2B-GFP, changing the temporal sampling rate at 3 s after the photobleach from 30 Hz to 0.5 Hz. As expected, after the switch to the slower sampling rate, the H2B-GFP FRAP curves showed a jump in the recovery curve (Fig. 4A). This jump was absent if the same sampling rate was used throughout the recovery. In contrast, a change in the sampling rate produced no such jump in H2B-Halo-TMR FRAP curves which were identical with or without the change in sampling rate (Fig. 4B). These results show that the TMR-HaloTag is insensitive to changes in temporal sampling rates during FRAP.

**Figure 5:**
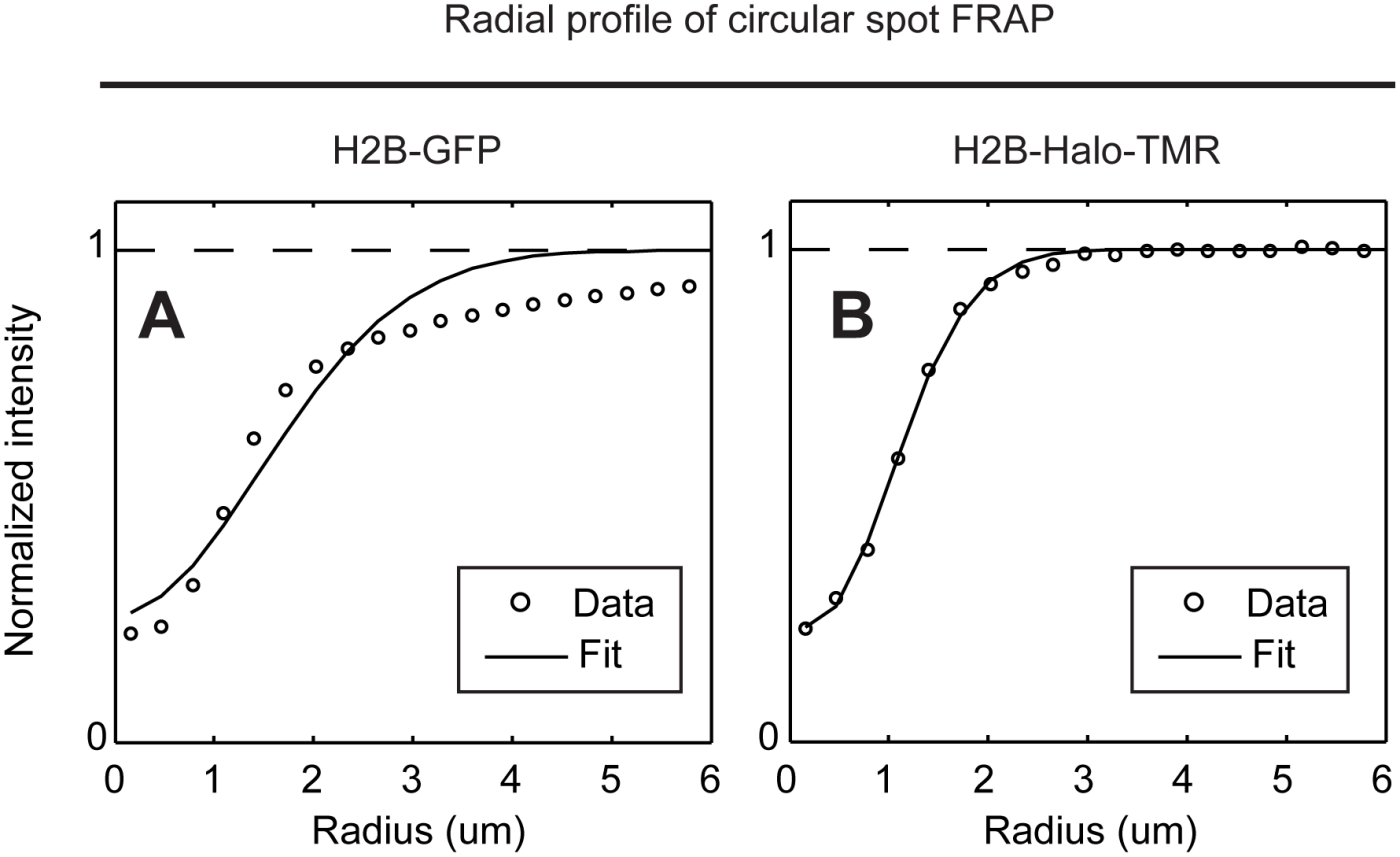
Artifacts in FRAP arising from alteration in the temporal sampling rate at the intentional photobleach. H2B spot FRAP was performed as in Fig. 2C, but now the photobleach profile immediately after the bleach was measured. With a GFP tag, the photobleach profile appears to extend a distance of up to 6 µm for a 0.5 µm radius bleach spot and could not be fitted with the conventional model for a photobleach profile [3] (A). This is an artifact due to an alteration in temporal sampling rates at the time of the photobleach imposed by our microscope software [9]. This effect is not observed with a TMR-HaloTag, where instead the photobleach profile returns to the normalized intensity of one within 3 um from the center of the photobleach and could be well fitted with the conventional model for a photobleach profile (B).

The fourth photoswitching artifact in FRAP was reported by Mueller et al. [9]. They found that the microscope software reduced the time interval between the last image before the bleach and the first image after the bleach. This caused the first image after the photobleach to be dimmer due to the disturbance in the photoswitching equilibrium that had been established during pre-bleach imaging. Mueller et al. showed that this disruption had a negligible effect on the FRAP curve itself, but they found it had a significant effect on the measured photobleaching profile. This profile is required as an initial condition to fit FRAP data with any form of quantitative model. In the presence of this photoswitching artifact, Mueller et al. found that the photobleaching profile no longer returned to a normalized value of one at the edge of the photobleach. We confirmed this result by photobleaching of H2B-GFP using a microscope whose software fails to maintain an equal temporal sampling rate at the time of the photobleach (Fig. 5A), and then showed that no such effect arose after photobleaching of H2B-Halo-TMR on the same microscope (Fig. 5B). This indicates that the TMR-HaloTag is not subject to this particular photoswitching artifact, and so is well suited to a quantitative FRAP analysis.

**Figure 6:**
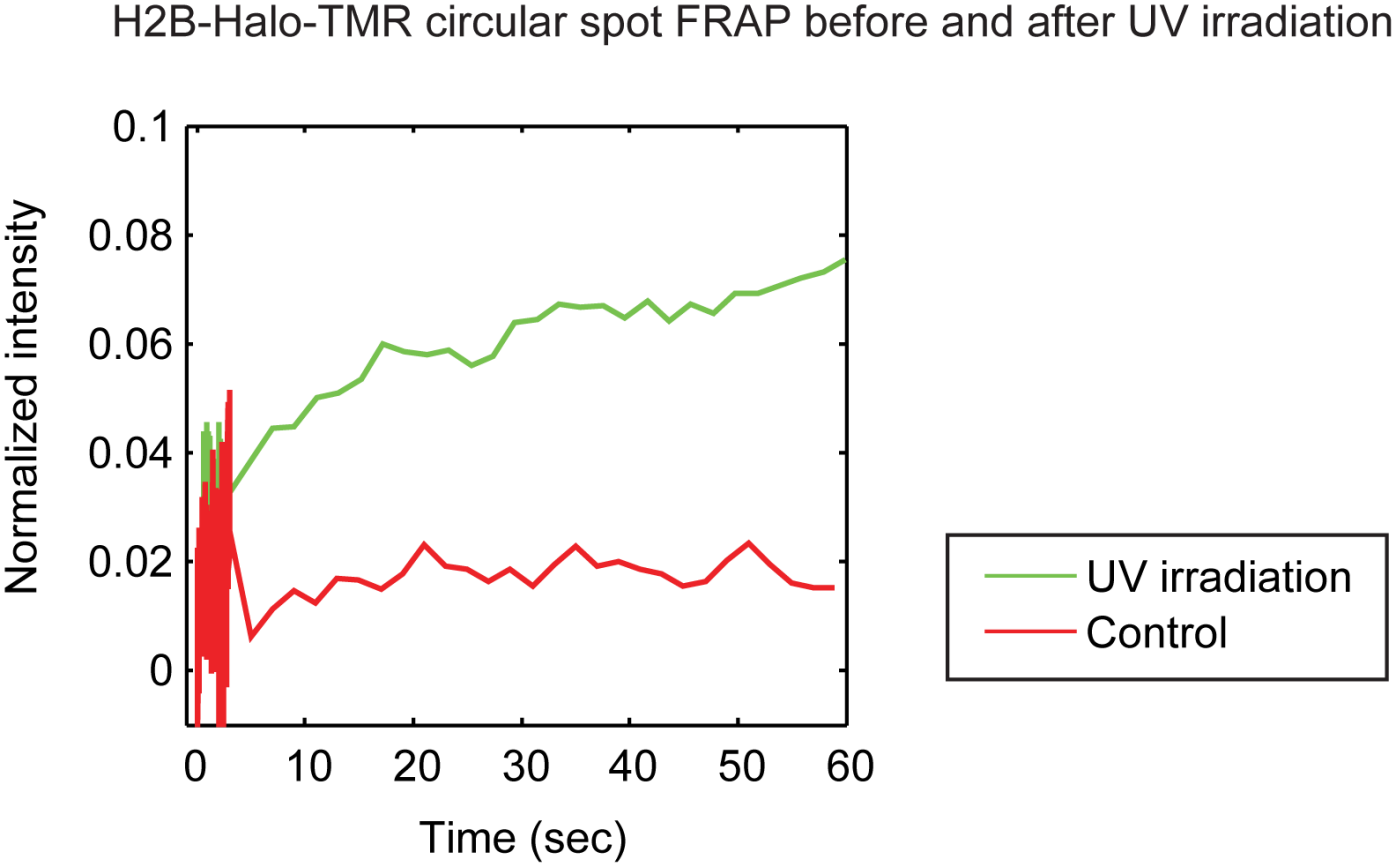
The dynamics of H2B-Halo-TMR with and without UV irradiation. Consistent with a previous study [19], the slow recovery phase of H2B after UV irradiation is faster than the control without UV irradiation. However, we could detect no significant difference in the size of the fast component between with or without UV irradiation suggesting that the free pool of H2B is not altered by UV irradiation.

Finally we applied this photoswitching-free FRAP procedure to investigate whether nucleosomes are lost from DNA following DNA damage. We performed FRAP of H2B-Halo-TMR within cells before and after UV irradiation. Consistent with a previous study [19], we found that the FRAP recovery of H2B is faster following UV irradiation (Fig. 6). However we did not observe a significant difference in the H2B fast component before and after UV irradiation (1.6±1.2% vs 3.0±3.0%). This indicates that UV damage does not significantly alter the free fraction of H2B, suggesting that nucleosomes are retained on the DNA following DNA damage by UV irradiation.

## Discussion

Photoswitching can be a serious problem in FRAP. This is especially true in cellular imaging, where GFP, the most widely used fluorophore for such FRAPs, can exhibit considerable photoswitching. This has led to artifacts ranging from significant overestimates of the free fraction of a protein [7], [9] to false identification of anomalous diffusive behavior [10]. Thus it would be desirable to find substitutes for GFP, (namely genetically encoded fluorophores) that would minimize the deleterious effects of photoswitching in cellular FRAP experiments. Our results here recommend TMR as such a substitute, as we found that under our conditions it could virtually eliminate all reported photoswitching artifacts in FRAP.

First, we found that the intentional photobleaching produced a negligible photoswitched fraction of H2B-Halo-TMR. In contrast, H2B-GFP yielded a 6.5–16% photoswitched fraction, with the magnitude of the photoswitched fraction increasing with weaker photobleaches, consistent with previous observations [9]. We then performed conventional spot FRAP of those H2B constructs. We found that FRAP of H2B-Halo-TMR produced a very small fast component (∼1%), whereas FRAP of H2B-GFP yielded ∼15% apparent fast component. We had earlier reported that these artifactual fast components in FRAPs of H2B-GFP could be corrected using an elaborate mathematical correction scheme [9]. This correction yielded an estimated fast component for H2B of ∼1%, regardless of the initial size of the artifactual fast component. Significantly, we now directly measured a similar fast component for H2B using TMR. These results validate the mathematical correction procedure and also demonstrate that TMR is immune to this photoswitching artifact. This property of TMR is especially valuable for proteins that exhibit slower FRAP recoveries, since for these proteins a substantial fraction of photoswitched molecules remain in the bleached region long enough to undergo photoswitching and thereby introduce a significant artifactual fast component.

Second, we showed that the TMR FRAP curve did not change after correction for observational photobleaching regardless of whether the intentional photobleach was performed early (only 1 or 2 pre-bleach images) or late (30 or 60 pre-bleach images). This means that TMR FRAPs can be performed with only a few pre-bleach images, which is an advantage for light sensitive specimens where 30–60 pre-bleach images would be detrimental. It is also an advantage for point FRAP experiments, where the photobleach is a diffraction limited spot. Here continued influx of fluorescence into the small spot prevents reaching the photoswitching equilibrium when a photoswitchable probe like GFP is used, and so the overcorrection of the FRAP curve after observational-photobleaching correction is unavoidable [10].

A third photoswitching artifact is the “blip” in the recovery curve when the temporal sampling rate is changed [7], [8]. We showed that TMR does not exhibit this artifact. Thus, TMR enables a reduction in temporal sampling rates during the slower phase of the FRAP recovery, which is often advantageous because it can reduce photodamage and photobleaching. Fourth, we found that the measured photobleach profile for TMR was not affected by the unequal temporal spacing of images before and after the photobleach often introduced by microscope software. This is important for quantitative FRAP analyses all of which require an accurate measurement of this photobleach profile [16].

Thus all of our data suggest that TMR exhibited virtually no photoswitchable fraction under typical FRAP imaging conditions. However, it should be noted that TMR does exhibit photoswitching under other imaging conditions [23]. Specifically, it has been used as a tag in dSTORM super-resolution microscopy, which explicitly depends on photoswitching. In dSTORM, the return of TMR to the bright state is greatly accelerated by switching the excitation light from 554 nm to 405 nm. This 405-catalyzed photoswitching is not likely to be a problem in TMR FRAP experiments which would, like our study here, only involve excitation at ∼550 nm. However, if there is any concern that different imaging and/or bleaching conditions might induce photoswitching of TMR, then those imaging conditions can be evaluated by performing a spot FRAP of H2B-Halo-TMR to confirm that the true fast component of ∼1% is measured.

Finally we applied this photoswitching-free FRAP to answer a biological question, namely if histone or nucleosome loss is involved in the chromatin opening which is known to occur after UV-induced DNA damage [17], [18]. Rather than wholesale nucleosome loss, one way to facilitate chromatin opening would be to simply accelerate the rate of exchange of some histone proteins with the damaged DNA. Indeed, evidence suggests that H2B exchange does increase after DNA damage [19], and our results here confirmed this observation. However, another way to facilitate chromatin opening following DNA damage would involve complete loss of some histone proteins or even loss of entire nucleosomes from the DNA. In either case, this should give rise to a larger free fraction of histones, which would most likely be reflected as a larger fast component in the FRAP recovery. We used H2B-Halo-TMR to measure the fast component of H2B before and after UV irradiation and found no significant difference. Thus our results provide the first evidence to suggest that H2B is not lost from nucleosomes after UV irradiation or that nucleosomes themselves are not lost from the DNA after UV irradiation. Instead, our results suggest that faster exchange of some histone proteins, such as H2B, is sufficient for the DNA repair process.

In sum, our results recommend TMR as a valuable label for FRAP experiments, since it appears to suffer none of the photoswitching artifacts that can confound the analysis of GFP FRAPs. Any protein of interest can be covalently labeled with TMR via the genetically encoded HaloTag fusion protein. The HaloTag protein is a 33 kDa protein that is increasingly used as a protein fusion tag in a wide variety of both in vivo imaging and in vitro biochemistry applications [21]. It should be noted that for established GFP fusion proteins and cell lines containing them, it is still possible to minimize the effects of GFP photoswitching either by fine tuning the bleach and imaging conditions or to largely eliminate the photoswitching artifacts by performing appropriate correction procedures [9]. However, for quantitative FRAP studies or for new qualitative FRAP analyses where fusion proteins are not yet constructed, the TMR-HaloTag approach should be carefully considered as it removes the risk of photoswitching artifacts which are otherwise often difficult to detect.

## Materials & Methods

### Cells and constructs

We used a previously described H2B-GFP [24] and H2B-Halo [25] constructs. Briefly, GFP is fused to C-terminus of histone H2B via a linker of 6 amino acids for H2B-GFP. HaloTag protein is fused to C-terminus of histone H2B via a linker of 17 amino acids derived from pFC15A (Promega) for H2B-Halo. For all FRAP experiments, HeLa cells were transiently transfected with H2B-GFP or H2B-Halo DNA using Lipofectamine LTX reagent (Invitrogen), according to the manufacturer’s instructions. At 24 hours after transfection, expressed H2B-Halo was labeled with HaloTag TMR ligand (Promega) according to the manufacturer’s instructions. As a control for whether the HaloTag protein might itself interact with chromatin, we performed FRAP of the unconjugated TMR-HaloTag protein in live cells and found equivalent recoveries to unconjugated GFP (Fig. S7 in Supporting Materials S1), which is not thought to exhibit significant interactions with chromatin or other cellular proteins (see Supporting Materials S1 Sect. 7 for details).

### Imaging conditions

All time-lapse experiments were performed on a Zeiss LSM 5 LIVE DuoScan confocal microscope using a 63X/1.4 NA oil immersion objective. The temperature of the incubated stage was set to 37°C, and the CO_2_ content was held at 5%. For each fluorophore, imaging size was 300×300 pixels (30×30 µm^2^) and imaging time was 33 ms with or without introducing the delay described in Fig. 1 of the Results. For imaging H2B-GFP, we used a 100 mW 488 nm line of a diode laser with BC488 beam splitter, NFT490 dichroic mirror and LP495 emission filter. For imaging H2B-Halo-TMR, we used a 40 mW 561 nm line of a DPSS laser with BC T25/R75 beam splitter, NFT 565 dichroic mirror and LP580 emission filter. Laser powers were determined such that comparable intensities were produced from either GFP or TMR (Fig. S4 in Supporting Materials S1). 6–10 nuclei were imaged and then the resultant fluorescent decay curves were averaged to produce a decay curve under each condition.

### FRAP conditions

All FRAP experiments were based on the imaging conditions described above. Typically, the laser power for photobleaching of GFP with the 488 nm line was 4.1 MW/cm^2^ and the laser power for photobleaching of TMR with the 561 nm line was 6.7 MW/cm^2^, except if noted otherwise in the figure legends. 6–10 FRAP curves were averaged to produce each FRAP curve for analyses.

## Supporting Information

Movie S1 **Representative movie of a whole nuclear photobleach of histone H2B.** The entire nucleus of HeLa cells expressing H2B-Halo-TMR (surrounded by a yellow line) was photobleached 4 sec after the start of image acquisition. The 4 sec of pre-bleach and the 20 sec of post-bleach images are shown at the same frame rate (30 Hz). There is virtually no recovery in fluorescence intensity inside the nucleus after the photobleaching. The corresponding fluorescence recovery curve is shown in Fig. 2B as a green curve.
(AVI)

Movie S2 **A representative movie of a circular spot FRAP of histone H2B.** A spot of 1.5 µm in diameter in a nucleus expressing H2B-Halo-TMR (surrounded by a yellow circle) was photobleached at 4 sec after starting the image acquisition. The 4 sec of pre-bleach and the 20 sec of post-bleach images are shown at the same frame rate (30 Hz). There is a very small fast recovery which is followed by a very slow recovery. The corresponding FRAP curve is shown in Fig. 2C and Fig. S6 as red curves.
(AVI)

Supporting Materials S1 **Data fitting and evaluations, kinetic model for photoswitching under imaging conditions, structural formula of TMR-HaloTag ligand, standard curve for excitation laser power for GFP and TMR, bleach depths of whole-nuclear photobleaches, H2B FRAP curves for 1 min and mobility of TMR-HaloTag protein and GFP in live cells.**
(DOCX)

## References

[1] Houtsmuller AB (2005) Fluorescence Recovery after Photobleaching: Application to Nuclear Proteins. In: Rietdorf J, editor. Microscopy Techniques. Advances in Biochemical Engineering. Springer Berlin Heidelberg. 177–199. Available: http://link.springer.com/chapter/10.1007/b102214. Accessed 26 February 2014. 10.1007/b102214 16080269

[2] Sprague BL, McNally JG (2005) FRAP analysis of binding: proper and fitting. Trends Cell Biol 15: 84–91 10.1016/j.tcb.2004.12.001 15695095

[3] Mueller F, Karpova TS, Mazza D, McNally JG (2012) Monitoring Dynamic Binding of Chromatin Proteins In Vivo by Fluorescence Recovery After Photobleaching. In: Morse RH, editor. Chromatin Remodeling. Methods in Molecular Biology. Humana Press. 153–176. Available: http://link.springer.com/protocol/10.1007/978-1-61779-477-3_11. Accessed 26 February 2014. 10.1007/978-1-61779-477-3_11 PMC7469828 22183594

[4] Owen DM, Williamson D, Rentero C, Gaus K (2009) Quantitative Microscopy: Protein Dynamics and Membrane Organisation. Traffic 10: 962–971 10.1111/j.1600-0854.2009.00908.x 19416480

[5] Catez F, Hock R (2010) Binding and interplay of HMG proteins on chromatin: Lessons from live cell imaging. Biochim Biophys Acta BBA - Gene Regul Mech 1799: 15–27 10.1016/j.bbagrm.2009.11.001 20123065

[6] Stasevich TJ, McNally JG (2011) Assembly of the transcription machinery: ordered and stable, random and dynamic, or both? Chromosoma 120: 533–545 10.1007/s00412-011-0340-y 22048163 PMC7380716

[7] Sinnecker D, Voigt P, Hellwig N, Schaefer M (2005) Reversible Photobleaching of Enhanced Green Fluorescent Proteins†. Biochemistry 44: 7085–7094 10.1021/bi047881x 15865453

[8] Bancaud A, Huet S, Rabut G, Ellenberg J (2010) Fluorescence Perturbation Techniques to Study Mobility and Molecular Dynamics of Proteins in Live Cells: FRAP, Photoactivation, Photoconversion, and FLIP. Cold Spring Harb Protoc 2010: pdb.top90 10.1101/pdb.top90 21123431

[9] Mueller F, Morisaki T, Mazza D, McNally JG (2012) Minimizing the Impact of Photoswitching of Fluorescent Proteins on FRAP Analysis. Biophys J 102: 1656–1665 10.1016/j.bpj.2012.02.029 22500766 PMC3318116

[10] Daddysman MK, Fecko CJ (2013) Revisiting Point FRAP to Quantitatively Characterize Anomalous Diffusion in Live Cells. J Phys Chem B 117: 1241–1251 10.1021/jp310348s 23311513

[11] Reits EAJ, Neefjes JJ (2001) From fixed to FRAP: measuring protein mobility and activity in living cells. Nat Cell Biol 3: E145–E147 10.1038/35078615 11389456

[12] Dayel MJ, Hom EFY, Verkman AS (1999) Diffusion of Green Fluorescent Protein in the Aqueous-Phase Lumen of Endoplasmic Reticulum. Biophys J 76: 2843–2851 10.1016/S0006-3495(99)77438-2 10233100 PMC1300255

[13] Dickson RM, Cubitt AB, Tsien RY, Moerner WE (1997) On/off blinking and switching behaviour of single molecules of green fluorescent protein. Nature 388: 355–358 10.1038/41048 9237752

[14] Henderson JN, Ai H, Campbell RE, Remington SJ (2007) Structural basis for reversible photobleaching of a green fluorescent protein homologue. Proc Natl Acad Sci 104: 6672–6677 10.1073/pnas.0700059104 17420458 PMC1871844

[15] Lemmer P, Gunkel M, Weiland Y, Müller P, Baddeley D, et al (2009) Using conventional fluorescent markers for far-field fluorescence localization nanoscopy allows resolution in the 10-nm range. J Microsc 235: 163–171 10.1111/j.1365-2818.2009.03196.x 19659910

[16] Mueller F, Wach P, McNally JG (2008) Evidence for a common mode of transcription factor interaction with chromatin as revealed by improved quantitative fluorescence recovery after photobleaching. Biophys J 94: 3323–3339 10.1529/biophysj.107.123182 18199661 PMC2275703

[17] Kruhlak MJ, Celeste A, Dellaire G, Fernandez-Capetillo O, Müller WG, et al (2006) Changes in chromatin structure and mobility in living cells at sites of DNA double-strand breaks. J Cell Biol 172: 823–834 10.1083/jcb.200510015 16520385 PMC2063727

[18] Soria G, Polo SE, Almouzni G (2012) Prime, Repair, Restore: The Active Role of Chromatin in the DNA Damage Response. Mol Cell 46: 722–734 10.1016/j.molcel.2012.06.002 22749398

[19] Dinant C, Ampatziadis-Michailidis G, Lans H, Tresini M, Lagarou A, et al (2013) Enhanced Chromatin Dynamics by FACT Promotes Transcriptional Restart after UV-Induced DNA Damage. Mol Cell 51: 469–479 10.1016/j.molcel.2013.08.007 23973375

[20] Kimura H, Cook PR (2001) Kinetics of Core Histones in Living Human Cells. J Cell Biol 153: 1341–1354 10.1083/jcb.153.7.1341 11425866 PMC2150718

[21] Urh M, Rosenberg M (2012) HaloTag, a Platform Technology for Protein Analysis. Curr Chem Genomics 6: 72–78 10.2174/1875397301206010072 23213345 PMC3480824

[22] Akaike H (1974) A new look at the statistical model identification. IEEE Trans Autom Control 19: 716–723 10.1109/TAC.1974.1100705

[23] Van de Linde S, Löschberger A, Klein T, Heidbreder M, Wolter S, et al (2011) Direct stochastic optical reconstruction microscopy with standard fluorescent probes. Nat Protoc 6: 991–1009 10.1038/nprot.2011.336 21720313

[24] Phair RD, Scaffidi P, Elbi C, Vecerová J, Dey A, et al (2004) Global Nature of Dynamic Protein-Chromatin Interactions In Vivo: Three-Dimensional Genome Scanning and Dynamic Interaction Networks of Chromatin Proteins. Mol Cell Biol 24: 6393–6402 10.1128/MCB.24.14.6393-6402.2004 15226439 PMC434243

[25] Mazza D, Abernathy A, Golob N, Morisaki T, McNally JG (2012) A benchmark for chromatin binding measurements in live cells. Nucleic Acids Res 40: e119–e119 10.1093/nar/gks701 22844090 PMC3424588

