# Supplementary material for: Photoswitching-Free FRAP Analysis with a Genetically Encoded Fluorescent Tag
Source: PLoS One. 2014 Sep 18;9(9):e107730. doi: 10.1371/journal.pone.0107730 (PMC4169462; doi:10.1371/journal.pone.0107730)
Supplement: Supporting Materials S1 — Data fitting and evaluations, kinetic model for photoswitching under imaging conditions, structural formula of TMR-HaloTag ligand, standard curve for excitation laser power for GFP and TMR, bleach depths of whole-nuclear photobleaches, H2B FRAP curves for 1 min and mobility of TMR-HaloTag protein and GFP in live cells. (DOCX) [file pone.0107730.s003.docx]

**Supplementary Information**

**1) Fits to GFP and TMR fluorescent decay curves with a line, a single exponential and a double exponential model for photoswitching**

In order to measure the decay kinetics of GFP and TMR during time lapse, we fit the fluorescent decay curves of GFP and TMR with a line ($I\left( t \right)=-kt+1$), a single exponential model ($I(t)=e^{-kt}$) and a double exponential model for photoswitching (see the next section for details of the photoswitching model). According to the model of Sinnecker et al. [1], a single exponential is expected for one irreversible photoconversion, namely photobleaching with the rate constant $k$, while a double exponential is expected for photoswitching with conversion rate $k_{1}$, reversion rate $k_{2}$ and photobleaching rate $k_{3}$.

To quantitatively evaluate fluorescent decay curves of GFP and TMR, we introduced the Akaike Information Criterion (AIC) [2]. The AIC can be used to distinguish between different fitting models and also quantify how much more likely one model is compared to another model. The AIC is defined by the equation below, where $N$ is the number of data points, $K$ is the number of parameters fit by the regression plus one, and $SS$ is the residual sum of squares.

$$AIC=N\cdot\ln\left( \frac{SS}{N} \right)+2K$$

The model with the lower AIC score is the model more likely to be correct. The relative likelihood (or evidence ratio) between two models can be calculated by the equation below, where $\Delta$ is the difference between AIC scores.

$$Evidence ratio=\frac{1}{e^{-0.5\Delta}}$$

By eye, we found that the fluorescent decay curves of GFP were poorly fit with a line and a single exponential but well fit with the double exponential model of Sinnecker et al. which they proposed to describe photoswitching (Fig. S2A). On the other hand, the fluorescent decay curves of TMR appeared to be reasonably fit with a line, a single exponential and the Sinnecker double exponential model (Fig. S2B). Applying the AIC to each of these fits, we found as expected that the Sinnecker double exponential model was the most likely explanation for the GFP fits (Table S1A), and that a single exponential model was the most likely explanation for the TMR fits (Table S1B).


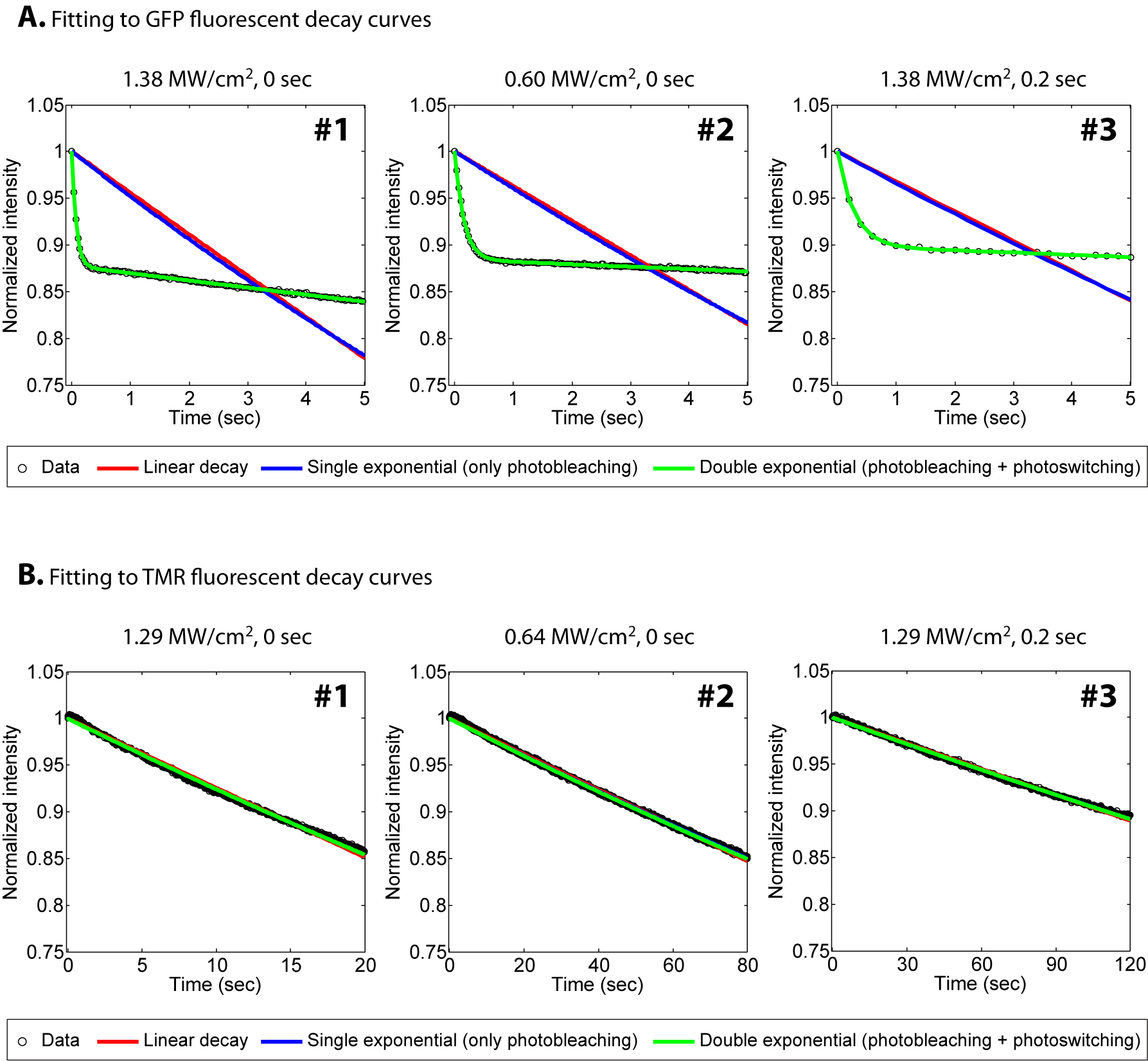


***Figure S1****. Fluorescent decay curves of H2B-GFP (A) and H2B-Halo-TMR (B) during time lapse imaging of entire nuclei in live cells. Red curves indicate linear fits to the data. Blue curves indicate fits to the data with a single exponential decay model which considers only irreversible photobleaching. Green curves indicate fits to the data with a double exponential model for photoswitching based on the interconversion scheme shown in Fig. 1C.* *Note that the decay curves up to 5 seconds were used for GFP fitting while decay curves up to 20, 80 and 120 seconds were used for TMR fitting in order that all of the curves showed a comparable loss in fluorescence (~15%).*

|  |  | | AIC score | | | Evidence ratio | |
| --- | --- | --- | --- | --- | --- | --- | --- |
| # | Laser power (MW/cm^2^) | Delay (sec) | Line | 1-exp | 2-exp | 2-exp / line | 2-exp / 1-exp |
| 1 | 1.38 | 0 | -853 | -865 | -2163 | 2.7 x 10^284^ | 5.7 x 10^281^ |
| 2 | 0.6 | 0 | -886 | -896 | -2164 | 3.0 x 10^277^ | 2.2 x 10^275^ |
| 3 | 1.38 | 0.2 | -161 | -163 | -365 | 2.2 x 10^44^ | 9.7 x 10^43^ |

***Table S1A.*** *AIC scores calculated for fits to GFP fluorescent decay curves. Evidence ratio quantifies which model is more likely to be correct. For example, under the condition #1, a double exponential model for photoswitching is 2.7 x 10^284^ times more likely to be correct than a linear model and is 5.7 x 10^281^ times more likely to be correct than a single exponential model.*

+

|  |  | | AIC score | | | Evidence ratio | |
| --- | --- | --- | --- | --- | --- | --- | --- |
| # | Laser power (MW/cm^2^) | Delay (sec) | line | 1-exp | 2-exp | 1-exp / line | 1-exp / 2-exp |
| 1 | 1.29 | 0 | -6903 | -7350 | -7346 | 1.3 x 10^97^ | 7.4 |
| 2 | 0.64 | 0 | -30058 | -31675 | -31341 | Inf | 3.3 x 10^72^ |
| 3 | 1.29 | 0.2 | -7451 | -7756 | -7752 | 1.6 x 10^66^ | 8.0 |

***Table S1B.*** *AIC scores calculated for fits to TMR fluorescent decay curves. Evidence ratio quantifies which model is more likely to be correct. For example, under the condition #1, a single exponential model for is 1.3 x 10^97^ times more likely to be correct than a linear model and is 7.4 times more likely to be correct than a double exponential model for photoswitching. Note, the evidence ratios against a double exponential model for photoswitching are relatively low under the condition #1 and #3, but the estimates for* $k_{1}$ *(the conversion rate into the dark state) were negligible (1.4 x 10^-4^ and 1.9 x 10^-7^, respectively) and the estimates for* $k_{2}$ *(the reversion rate from the dark state) were extremely large (6.6 x 10^3^ and 1.5 x 10^3^, respectively). In addition, the estimates for* $k_{3}$ *(the irreversible photobleaching rate) were identical to that determined from the single exponential fit* $k$ *(0.0079 and 0.0010, respectively). These results suggest that TMR has at best a negligible amount of photoswitching under these imaging conditions.*

**2)** **Kinetic model for photoswitching under imaging conditions**

Sinnecker et al. [1] derived a simple kinetic model which describes the typical fluorescence decay observed for GFP and its derivatives under illumination conditions. Although their study described the model, they did not present an explicit solution. Here we summarize their model and write out the specific biexponential decay predicted by their model.

The kinetic model of Sinnecker et al. [1] considers three different states: the native, fluorescent bright state ${FP}_{nat}$, a photoswitched dark state ${FP}_{swi}$, and an photobleached state ${FP}_{ble}$. The transition between these states is described by three different rates constants: the reversible transition between ${FP}_{nat}$and ${FP}_{swi}$ is characterized by the rate constants $k_{1}$ and $k_{2}$, and the irreversible transition from ${FP}_{nat}$ to ${FP}_{ble}$ by $k_{3}$ (Fig 1C). Sinnecker et al. assume first-order kinetics for all transitions which yields the following system of differential equations (Eq. 1):

$$\frac{d\left[ {FP}_{nat} \right]}{dt}= -\left( k_{1}+k_{3} \right)\left[ {FP}_{nat} \right]+k_{2}\left[ {FP}_{swi} \right]$$

$$\frac{d\left[ {FP}_{swi} \right]}{dt}= -k_{2}\left[ {FP}_{swi} \right]+k_{1}[{FP}_{nat}]$$

Assuming initial conditions $\left[ {FP}_{nat} \right]=1, \left[ {FP}_{swi} \right]=0, \left[ {FP}_{ble} \right]=0$, this set of equations can be solved with standard methods to obtain (Eq. 2):

$$\left[ {FP}_{nat} \right]\left( t \right)=\frac{1}{2w}\left( \left( k_{1}-k_{2}+k_{3}+w \right)e^{\frac{1}{2}\left( -v-w \right)t}+\left( -k_{1}+k_{2}-k_{3}+w \right)e^{\frac{1}{2}\left( -v+w \right)t} \right)$$

$$v=k_{1}+k_{2}+k_{3}$$

$$w=\sqrt{\left( k_{1}+k_{2}+k_{3} \right)^{2}-4k_{2}k_{3}}$$

To fit time-lapse data with the preceding model, it should be renormalized such that the first time-point is 1. The resulting curve can then be fit with Eq. 2 and rate constants $k_{1}, k_{2}$ and $k_{3}$ can be extracted.

**3) Structural formula of TMR-HaloTag ligand**

**
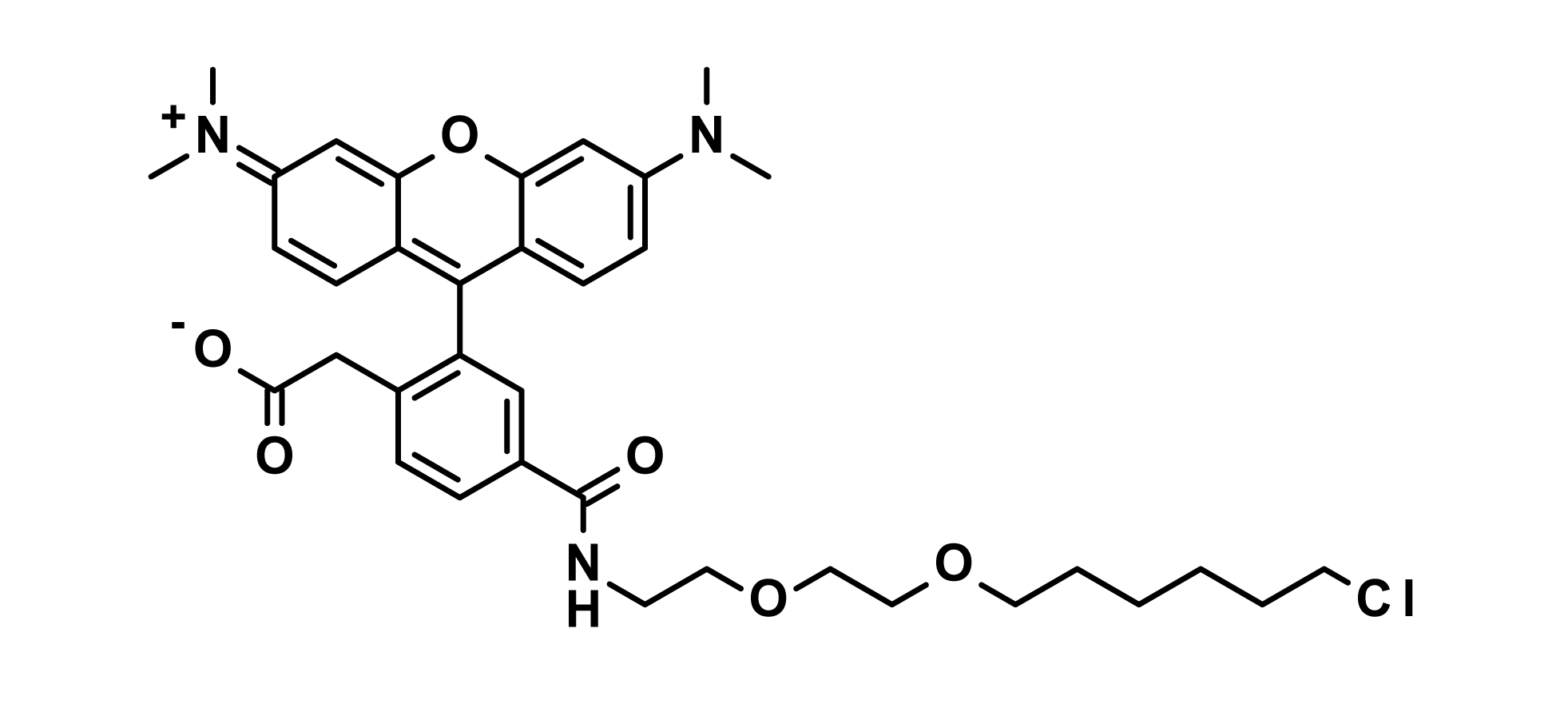
**

**4) Standard curve for excitation laser power for GFP and TMR**

Photoswitching behavior is very sensitive to the excitation laser power. For a fair comparison of the photoswitching properties of GFP and TMR we excited each fluorophore at laser powers which yielded equivalent amounts of fluorescence.

To determine these equivalent laser powers, we first measured the fluorescent intensity of GFP and TMR in the nucleus at a series of different excitation laser powers. The resultant data for each fluorophore were then fit with a line which related laser excitation level with fluorophore intensity (Fig S4A and S4B). By equating ${GFP}_{int}$ and ${TMR}_{int}$ we solved for the linear relationship between ${GFP}_{ex}$ and ${TMR}_{ex}$ (Fig. S4C). This line was then used to find equivalent laser powers.


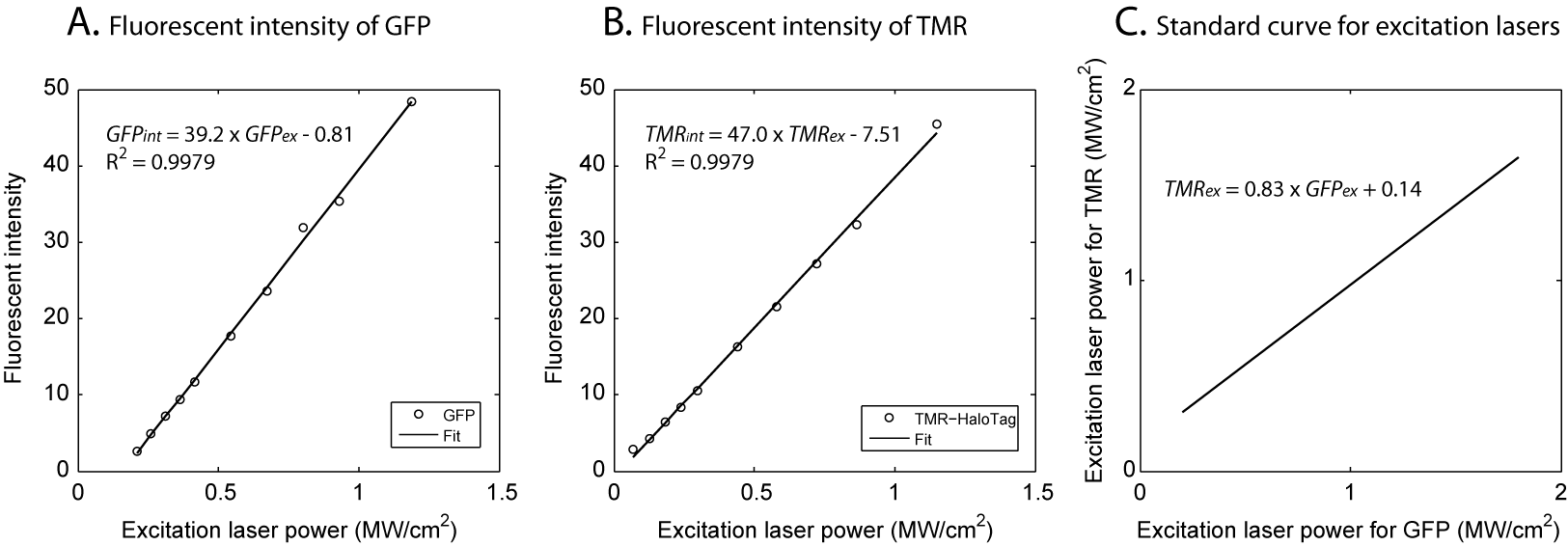


***Figure S4****. Relationship between fluorescent intensity and laser power. The fluorescent intensities of both GFP and TMR in the nucleus increased linearly with excitation laser power (A and B). The fits to these data were used to calculate a standard curve defining equivalent laser excitation powers for GFP and TMR (C).*

**5) Bleach depths of whole-nuclear photobleaches**

For a fair comparison, the laser power to bleach TMR was determined empirically such that the bleach depth of a TMR whole-nuclear photobleach was comparable to the bleach depth of a GFP whole-nuclear photobleach.


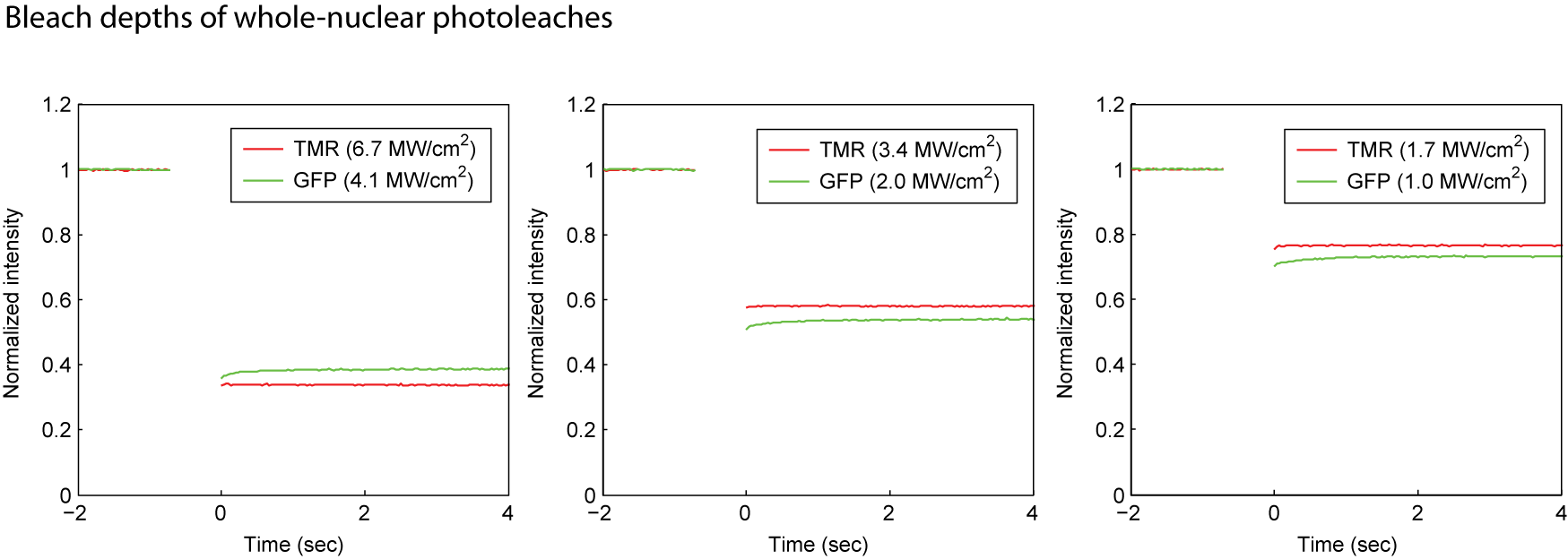


***Figure S5****. Shown are three different bleach depths with the corresponding laser powers used to achieve these different bleach depths.*

**6) Circular spot FRAP of H2B**


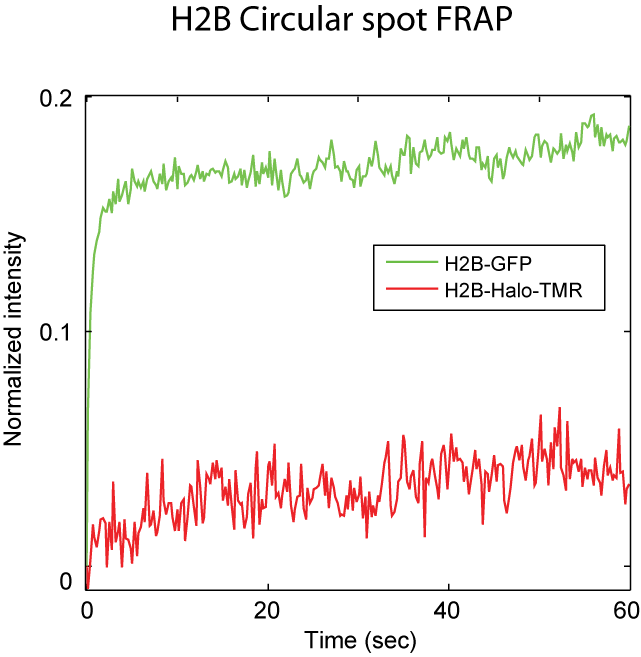
Fig. 2C in the main text shows a comparison of H2B-GFP and H2B-Halo-TMR FRAP recoveries on a short time scale in order to highlight the difference in the fast component. This comparison showed that the GFP FRAP had a much larger, artifactual fast component. Here we compare the same two FRAP curves but now on a much longer time scale. This demonstrates that the slow component of the FRAP is similar for GFP and TMR, as the two curves are parallel over a long time scale. Note that the TMR signal is noisier than the GFP signal. This is because the GFP signal is artifactually brighter than the TMR signal due to the large photoswitching fraction of GFP.

***Figure S6.*** *FRAP curves of H2B-GFP and H2B-Halo-TMR for 1 minute. The curves for the first 5 seconds are shown in Fig. 2C.*

**7) Mobility of TMR-HaloTag protein and GFP in live cells**


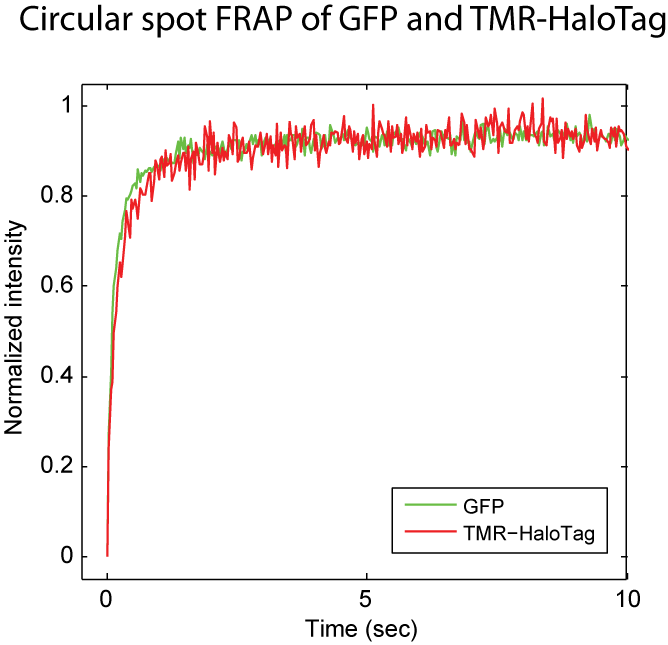
We tested whether the TMR-HaloTag protein by itself might interact with chromatin or other proteins in cell nuclei, and therefore be an unfavorable label for proteins. We tested this by performing FRAP of the TMR-HaloTag protein and comparing it to FRAP of GFP under identical conditions. We found that both proteins recovered identically suggesting that the HaloTag is suitable for studying the mobility of nuclear proteins.

***Figure S7****. FRAP curves of GFP and TMR-HaloTag protein in live cells. FRAP experiments were performed on cells expressing either GFP or TMR labeled HaloTag proteins.*

**Supplementary Movies**

Supplementary Movie 1: Representative movie of a whole nuclear photobleach of histone H2B. The entire nucleus of HeLa cells expressing H2B-Halo-TMR (surrounded by a yellow line) was photobleached 4 sec after the start of image acquisition. The 4 sec of pre-bleach and the 20 sec of post-bleach images are shown at the same frame rate (30 Hz). There is virtually no recovery in fluorescence intensity inside the nucleus after the photobleaching. The corresponding fluorescence recovery curve is shown in Fig 2B as a green curve.

Supplementary Movie 2: A representative movie of a circular spot FRAP of histone H2B. A spot of 1.5 µm in diameter in a nucleus expressing H2B-Halo-TMR (surrounded by a yellow circle) was photobleached at 4 sec after starting the image acquisition. The 4 sec of pre-bleach and the 20 sec of post-bleach images are shown at the same frame rate (30 Hz). There is a very small fast recovery which is followed by a very slow recovery. The corresponding FRAP curve is shown in Fig. 2C and Fig. S6 as red curves.

**References**

1. Sinnecker D, Voigt P, Hellwig N, Schaefer M (2005) Reversible Photobleaching of Enhanced Green Fluorescent Proteins†. Biochemistry 44: 7085–7094. doi:10.1021/bi047881x.

2. Akaike H (1974) A new look at the statistical model identification. IEEE Trans Autom Control 19: 716–723. doi:10.1109/TAC.1974.1100705.
